# Late sacral recurrence of rectal cancer treated by heavy ion radiotherapy: a case report

**DOI:** 10.1186/s40792-016-0240-8

**Published:** 2016-10-10

**Authors:** Hiroyuki Matsuzaki, Soichiro Ishihara, Kazushige Kawai, Takeshi Nishikawa, Toshiaki Tanaka, Tomomichi Kiyomatsu, Keisuke Hata, Hiroaki Nozawa, Shigeru Yamada, Toshiaki Watanabe

**Affiliations:** 1 Division of Surgical Oncology, Department of Surgery, Faculty of Medicine, University of Tokyo, 7-3-1 Hongo, Bunkyo-ku, Tokyo, 113-8655 Japan; 2 Research Center Hospital for Charged Particle Therapy, National Institute of Radiological Sciences, 4-9-1 Anagawa, Inage-ku, Chiba-shi, Chiba 263-8555 Japan

**Keywords:** Heavy ion, Carbon ion, Bone metastasis, Late recurrence, Rectal cancer

## Abstract

**Background:**

The need for surveillance of rare late recurrence of rectal cancer has not yet been established. Local control of unresectable skeletal metastasis is important for palliation of symptoms and support for systemic chemotherapy.

**Case presentation:**

A Japanese man underwent preoperative pelvic irradiation (50.4 Gy/28 Fr) and low anterior resection at the age of 57 years. The pathological stage was II (T3N0M0). Nine years after the surgery, his carcinoembryonic antigen (CEA) level showed rapid elevation, although he had no symptoms. A computed tomography (CT) scan showed no evidence of recurrent lesions, but positron emission tomography (PET)-CT revealed abnormally high 2-[^18^F]-fluoro-2-deoxy-d-glucose accumulation in the sacrum. A CT-guided needle biopsy confirmed the diagnosis of metastatic adenocarcinoma from the previous rectal cancer. The sacral metastasis reached the S1/S2 level and was considered inoperable. Conventional radiotherapy was also excluded due to the previous history of pelvic irradiation. Finally, heavy ion radiotherapy with carbon ions was performed as radical local therapy (70.4 GyE/16 Fr). The patient did not consent to systemic chemotherapy immediately after the irradiation. Five months after radiotherapy, multiple lung metastases were noted on CT, followed by mediastinal and hilar lymph node metastases. Systemic chemotherapy was started 9 months after the irradiation. During this time, the patient experienced some degree of pain and loss in muscle strength of the left lower limb, and a second heavy ion irradiation (60.0 GyE/12 Fr) was performed 11 months after the previous irradiation. After that, the sacral lesion has been stable and his symptoms have not worsened. Two years after the heavy ion therapy, the patient steadily continues outpatient chemotherapy and his quality of life is relatively maintained.

**Conclusion:**

In case the risk of late recurrence is relatively high after rectal cancer surgery, clinicians should consider individual follow-up evaluations, including CEA measurements to allow for timely diagnosis and intervention. Heavy ion radiotherapy is effective for local control of sacral metastasis.

## Background

Most recurrences of colorectal cancer occur within 5 years after curative resection, and the guidelines recommend a 5-year postoperative follow-up [1, 2]; however, a few cases do show relapse beyond this time period, with symptomatic and sometimes advanced stages causing severe complications. Bone metastasis from colorectal cancer is a relatively rare event [3–6], but it is often accompanied by serious problems such as pain, paralysis, and pathological fractures; therefore, therapeutic intervention is required as early as possible. There is no established treatment for bone metastases from colorectal cancer, and palliative radiotherapy is sometimes used for pain control and prevention of pathological fracture. On the other hand, heavy ion radiotherapy using carbon ions has been reported to be effective for local recurrence of rectal cancer [7–9]. Here, we report a rare and interesting case of a late sacral recurrence treated with heavy ion radiotherapy 9 years after the curative resection of a primary rectal cancer.

## Case presentation

A 57-year-old man was referred to our hospital because of blood in the stool, and he was diagnosed with clinical stage II (T3N0M0) rectal cancer in 2004. The preoperative carcinoembryonic antigen (CEA) level (8.1 ng/ml) was slightly above the normal range (0–5.0 ng/ml). Preoperative pelvic irradiation with 50.4 Gy/28 Fr was performed, and the tumor decreased in size. Low anterior resection was performed.

The resected specimen contained a 2.0-cm × 2.0-cm moderately differentiated adenocarcinoma with slight venous invasion and partial effect of irradiation and was diagnosed as stage II (T3N0M0) according to the Union for International Cancer Control TNM staging system. The surgical margin was negative. The blood CEA level dropped to the normal value soon after the operation. Adjuvant 5′-deoxy-5-fluorouridine was orally administered for 2 years after the operation. The follow-up examinations showed no sign of recurrence, and 5 years after the surgery, the follow-up was continued with annual computed tomography (CT) scans and biannual blood tests, including CEA measurements.

**Fig. 1:**
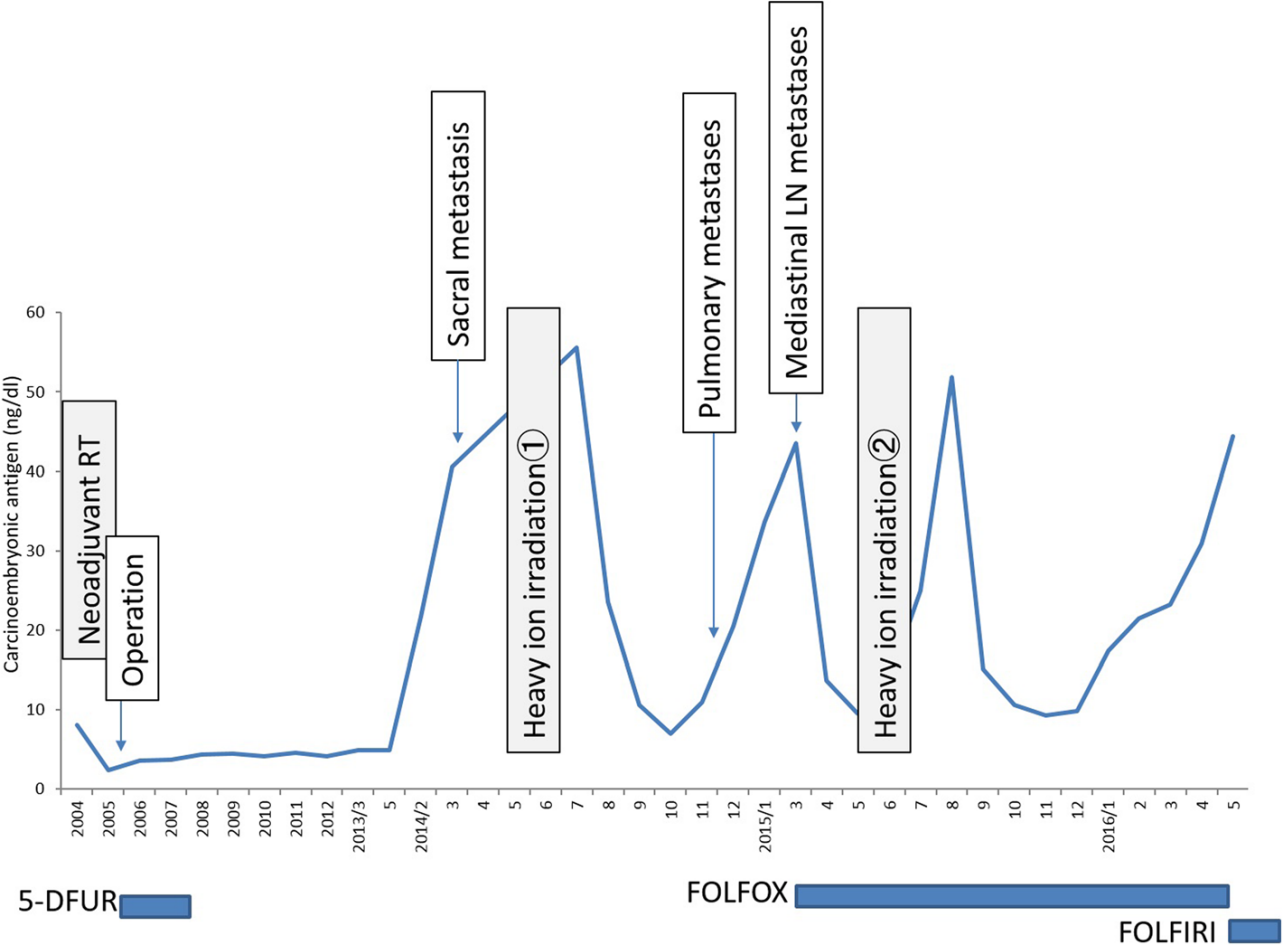
Course of treatment and changes in blood CEA level

**Fig. 2:**
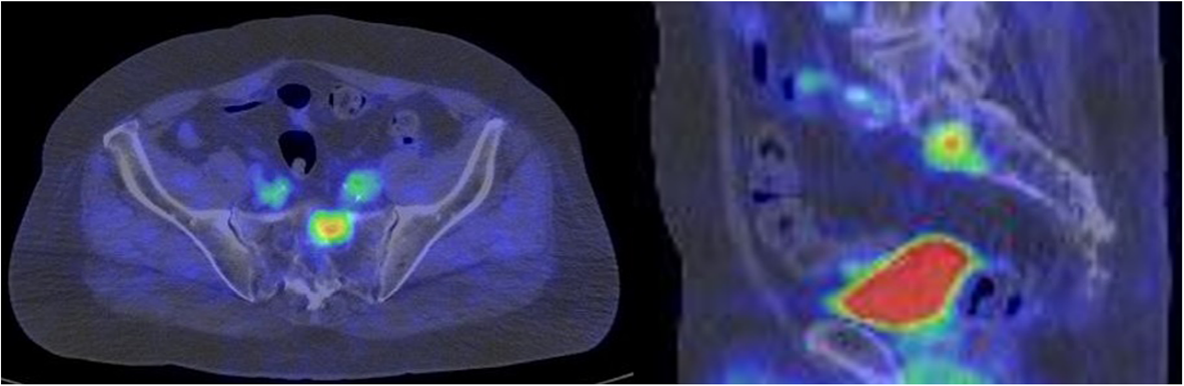
FDG-PET showing sacral recurrence 9 years after the surgery on the primary tumor. FDG-PET-CT shows abnormally high accumulation of FDG in the first and second segments of the sacrum 9 years after resection of the primary rectal cancer

Nine years after the surgery, the CEA level showed a rapid elevation to 21.7 ng/ml, although the patient had no symptoms (Fig. 1). A CT scan showed no evidence of recurrent lesions, but a positron emission tomography (PET)-CT scan revealed abnormally high 2-[^18^F]-fluoro-2-deoxy-d-glucose (FDG) accumulation only in the sacrum (Fig. 2). A CT-guided percutaneous needle biopsy of the sacral tumor confirmed the diagnosis of metastatic adenocarcinoma from the previous rectal cancer. The sacral metastatic lesion reached the left S1/S2 level, and the possibility of surgery was excluded to avoid nerve injury. Conventional radiotherapy was also excluded due to the previous history of irradiation to the pelvis. The primary rectal lesion had a *RAS* mutation.

Finally, heavy ion irradiation with carbon ions was performed as radical local therapy (70.4 GyE/16 Fr). The irradiation was performed as one of the “advanced medical treatments,” stipulated by the Ministry of Health, Labour and Welfare in Japan, after approval by the hospital case review committee. Written and informed consent was obtained from the patient. The dose was calculated for the target volume and any nearby critical structures and expressed in gray equivalent (GyE = carbon physical dose (Gy) × relative biologic effectiveness (RBE)). The CEA level reached a maximum of 55.6 ng/ml immediately after the heavy ion therapy and then gradually decreased to a minimum of 7.0 ng/ml 3 months after the irradiation.

**Fig. 3:**
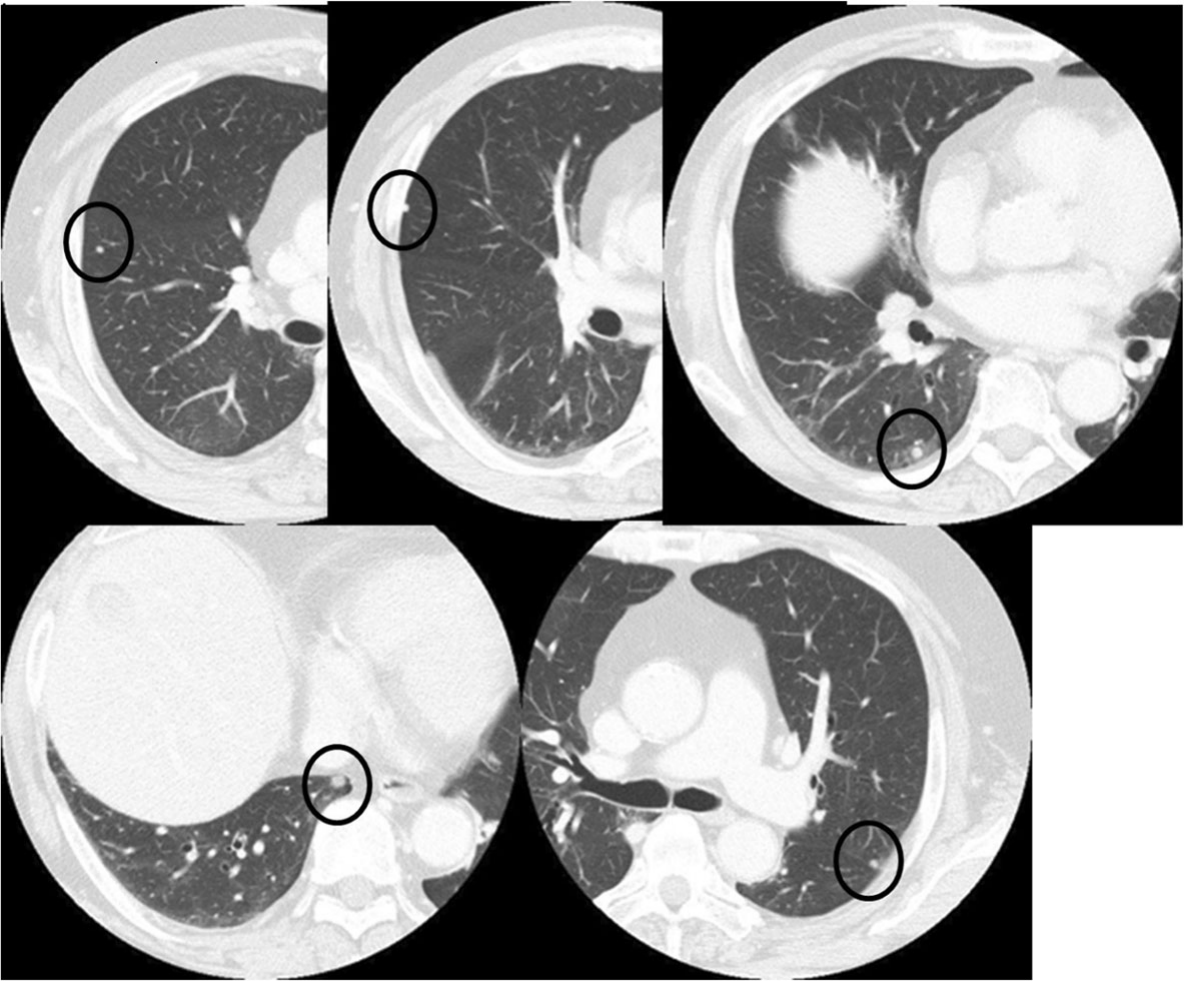
Chest CT 5 months after heavy ion radiotherapy. The chest CT shows multiple lung metastases 5 months after the heavy ion radiotherapy (*circles*)

**Fig. 4:**
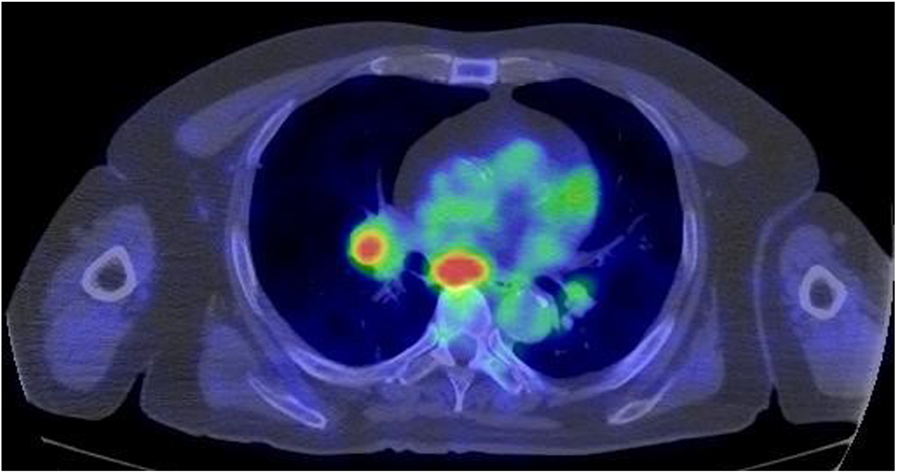
FDG-PET 9 months after heavy ion radiotherapy. PET-CT shows mediastinal and hilar lymph node metastases 9 months after the heavy ion radiotherapy

Considering the possibility of distant metastasis, the option of adjuvant systemic chemotherapy was presented but rejected by the patient. Four months after the heavy ion therapy, the CEA level was elevated again to 11.0 ng/ml and continued to increase thereafter. Multiple small lung metastases were noted on CT scans 5 months after the radiotherapy (Fig. 3), but the sacral lesion was stable. At first, resection of the lung metastases was considered, but this was eventually decided against because the PET-CT scan showed the appearance of mediastinal and hilar lymph node metastases 8 months after the radiotherapy (Fig. 4).

Systemic chemotherapy (mFOLFOX6 + bevacizumab) was started 9 months after the heavy ion radiotherapy. After three courses of the regimen, the patient developed acute hemorrhagic cholecystitis and was treated conservatively. Around the same time, he developed a decrease in left lower limb muscle strength and needed a wheelchair or walking brace when going out. Furthermore, left limb pain increased and pain control was started with oral administration of tapentadol, pregabalin, and oxycodone. Around the same time, a follow-up CT scan revealed asymptomatic pulmonary arterial and left lower limb deep vein thrombosis, and an oral anticoagulant was started. These bleeding and thrombosis events were thought to be side effects of bevacizumab, which was stopped thereafter.

**Fig. 5:**
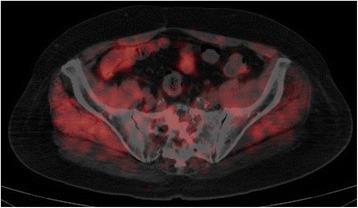
PET-CT 2 months after the second course of heavy ion radiotherapy. PET-CT shows disappearance of the abnormal accumulation in the sacral lesion 2 months after the second course of heavy ion radiotherapy

Eleven months after the irradiation, the sacral lesion showed slight progression with worsening of pain, and the second course of heavy ion irradiation (60.0 GyE/12 Fr) was administered. After the irradiation, the sacral lesion remained stable (Fig. 5) and there was no further worsening of pain. Two years after the first heavy ion irradiation, mFOLFOX6 has been continued for 25 courses and the regimen is planned to be changed to FOLFIRI because of the progression of both the lung metastases and the metastases in the mediastinal lymph nodes. There is no sign of further progression of the sacral lesion. The patient steadily continues outpatient chemotherapy without further worsening of the symptoms. His quality of life is relatively maintained; he travels abroad in a wheelchair once every few months with the help of his wife, after the administration of pain control drugs.

### Discussion

Most recurrences of colorectal cancer occur within 5 years after curative surgery, and the guidelines in America and Japan recommend a 5-year postoperative follow-up [1, 2]. However, a few cases do show relapse after an interval of more than 5 years, and there is no recommendation on surveillance for such possible late recurrences. Some risk factors of late recurrence have been reported, including rectal location of the cancer, early stage at diagnosis, neoadjuvant (chemo)radiation therapy for rectal cancer, pathologically well-differentiated disease, and low venous invasion [10–13]. Merkel et al. recommended at least 7–8 years of follow-up after long-term neoadjuvant radiotherapy or chemoradiotherapy for rectal cancer, because of the high rate of local recurrences noted more than 5 years after the primary therapy [10]. With respect to the mechanism involved in the late recurrence in rectal cancer patients who had received long-term neoadjuvant (chemo)radiation therapy, our literature search did not reveal any convincing results.

In the case we report here, the patient had received preoperative radiotherapy and therefore had a potential risk of late recurrence. Under such a condition, a rapid elevation of the CEA level led to further investigations to identify metastatic lesions. If the follow-up had been terminated at the 5-year limit, the sacral metastasis would not have been diagnosed until the patient developed symptoms due to the metastatic lesion. Although CEA measurement is widely performed in the postoperative follow-up of colorectal cancer for the detection of asymptomatic recurrence, there is no established recommendation about how often or until when CEA levels should be monitored [14–16]. For relatively young and healthy patients who can be candidates for radical therapy if metastatic lesions are found, annual or biannual CEA measurements may be a good choice for the detection of late recurrence, in case the assumed recurrence risk is relatively high. Of course, ongoing evaluation is necessary of the effectiveness of CEA measurement, including long-term efficacy and cost.

FDG-PET has been reported to be useful in the diagnosis of recurrent cancer, including colorectal cancer [17–19]. Flanagan et al. reported that, in 22 colorectal cancer patients with abnormal postoperative CEA levels but negative results on conventional imaging studies, the positive predictive value of FDG-PET was 89 % (15 of 17) and the negative predictive value was 100 % (5 of 5) [20]. In our case, a conventional CT scan could not show the metastatic lesion, even when examined retrospectively, while PET-CT could detect abnormally high FDG accumulation in the sacrum. Therefore, we conclude that PET-CT should be performed to search for metastatic or recurrent lesions undetectable by other imaging tests.

As there are no definitive or routine follow-up schedules for late recurrence, we should consider tailoring them based on each individual case. For relatively young and healthy patients who may be candidates for radical therapy for metastatic lesions, annual or biannual CEA measurements/digital examination/(PET-) CT may be appropriate choices for the detection of late recurrence. CEA measurement and digital examination may be ideal since they are easy to perform with relatively low cost and low invasiveness. In terms of ceasing follow-up, 7–10 years may be sufficient, although there is no supporting evidence. Merkel et al. recommend at least 7–8 years of follow-up after long-term neoadjuvant radiotherapy or chemoradiotherapy [10]. However, in the current case, the recurrence occurred 9 years after the primary surgery. It is important to take into consideration the patients’ age, comorbidity, activities of daily living, and hope for intensive follow-up. At any rate, we think routine termination of follow-up at 5 years may not always be appropriate for those patients with a potentially high risk of late recurrence.

Skeletal metastasis is relatively rare in colorectal cancer compared with the more common liver, peritoneal, and lung metastases. In previous case series, the rate of skeletal metastasis has been reported to be approximately 6 % [3, 4]. In recent decades, higher rates of bone metastasis have been documented in association with the administration of multiple systemic treatments [5, 6]. Sundermeyer et al. reported that 10.4 % of patients with metastatic colorectal carcinoma developed bone metastases, and the frequency of this event increased with the number of chemotherapy drugs used, from 3.7 % in cases of no systemic chemotherapy to 17.4 % in cases where four to five agents were used; they added that bone metastases develop more frequently in rectal cancer than in colon cancer [5]. Katoh et al. reported that 23.7 % (28/118) of autopsy cases with colorectal cancer had bone metastases [21]. In our case, the sacral metastasis would be from blood circulation including Batson’s venous plexus [22] because initial pathological findings showed no lymph node metastasis nor lymphatic invasion.

There is no established treatment for skeletal metastases from colorectal cancer. Conventional radiotherapy alone can hardly be considered a radical treatment but is still effective for the prevention and palliative treatment of symptoms from bone metastases, such as pain, paralysis, and pathological fracture. Surgical resection is considered when the metastasis is limited to a single bone site and is curable, if the associated risk of surgery is acceptable. For sacral metastasis, surgery is usually not indicated for S1/S2 lesions because of the high risk of neurological complications. Moriya et al. reported that, in 57 patients with locally recurrent rectal cancer who underwent total pelvic exenteration with distal sacrectomy, the most frequent complication was sacral wound dehiscence (51 % of patients), followed by pelvic sepsis (39 %), but serious complications such as walking impairment and spinal fluid leak did not occur since their guidelines included preservation of the S2 sacral nerves bilaterally [23]. Fourney et al. reported that, in 29 patients who underwent en bloc resection of primary sacral tumors, the rates of walking impairment when the level of sacrectomy included S1 and below S1 were 40 and 14 %, respectively, but no patients had walking impairment if the sacrectomy was below S2 [24].

In the current case, a sacrectomy was decided against because the metastatic lesion reached the S1/S2 level and the risk of nerve injury was high. Conventional radiotherapy was also not indicated because of the history of preoperative pelvic irradiation. On the other hand, heavy ion radiotherapy using carbon ions has been reported to produce good clinical results for local recurrence of rectal cancer [7–9]. Yamada et al. performed a phase I/II study in which 180 patients were treated with carbon ion radiotherapy for locally recurrent rectal cancer. In their study, no patients experienced acute reactions of National Comprehensive Cancer Network grade 3 to 5 severity; the local control rates in patients treated with 67.2, 70.4, and 73.6 GyE at 5 years were 35, 77, and 88 %, respectively; the overall survival rates in patients treated with 73.6 GyE were 78 % at 3 years and 59 % at 5 years [25]. They also reported another study in which 23 patients were treated with carbon ion radiotherapy (70.4 GyE) as re-irradiation for locally recurrent rectal cancer. In this study, grade 3 toxicities occurred in six (26 %) patients and the 1- and 3-year overall survival rates were 83 and 72 %, respectively [26]. Habermehl et al. also reported that, in 19 patients treated with carbon ion radiotherapy as re-irradiation of local recurrence of rectal cancer, the estimated mean local progression-free survival was 20.6 months with no grade 3 or higher toxicities [9].

To avoid the complication of bowel perforation from heavy ion therapy, the recurrent lesion must be separated from the intestinal wall by a space of at least 5 mm; our case met this criterion. If the spacing is not adequate, heavy ion radiotherapy can be performed safely by surgically inserting a spacer between the target lesion and the intestinal wall.

In the current case, pain and palsy occurred 1 year after the first course of heavy ion radiotherapy. At first, the nerve disorder may have been an adverse event as a result of the heavy ion radiotherapy. It generally occurs 6–12 months after irradiation, in a small number of people. Since this patient had undergone conventional neoadjuvant radiotherapy during the primary treatment, the accumulative dose to the nerve was high, which may be one of the causes of the nerve disorder. However, as for the worsening of pain prior to the second course of heavy ion radiotherapy, we do not think it was merely an adverse event; in fact, we think it was a symptom of sacral lesion progression. In fact, after the second heavy ion irradiation, further worsening of the nerve disorder ceased and we thought the irradiation was effective. If we had not intervened at the time of diagnosis, far more severe destruction of the sacrum and refractory pain might have occurred.

We performed a PubMed search of the literature using the keywords “heavy ion,” “carbon ion,” and “colorectal cancer” and found no reported case of heavy ion radiotherapy for bone metastasis from colorectal cancer. In our case, the heavy ion radiotherapy, which was potentially radical at the time of the irradiation, was unfortunately soon followed by the development of systemic metastases. Nevertheless, the sacral metastasis has been stable after the second heavy ion irradiation, and the patient’s quality of life has been relatively maintained until now. The patient steadily continues outpatient chemotherapy and enjoys his everyday life, including occasional foreign trips with his family. Quality of life is very important in cancer patients, especially those with advanced-stage disease. Heavy ion irradiation to the sacral lesion was considered effective for local control, continuation of systemic therapy, and eventually for maintenance of the quality of life.

## Conclusions

We encountered a rare case of late asymptomatic sacral recurrence of rectal cancer diagnosed by a rapid CEA level elevation 9 years after the primary surgery. Heavy ion irradiation to the sacral lesion was effective for local control. We conclude that, if the risk of late recurrence is expected to be relatively high, clinicians should consider individual follow-up evaluations, including CEA measurement, even beyond the 5-year limit, to allow timely diagnosis and intervention.

## Abbreviations

CEA: Carcinoembryonic antigen
CT: Computed tomography
FDG: 2-[^18^F]-fluoro-2-deoxy-d-glucose
GyE: Gray equivalent
PET: Positron emission tomography
